# *Eucalyptus* Tree: A Potential Source of *Cryptococcus neoformans* in Egyptian Environment

**DOI:** 10.1155/2016/4080725

**Published:** 2016-01-13

**Authors:** Mahmoud Elhariri, Dalia Hamza, Rehab Elhelw, Mohamed Refai

**Affiliations:** ^1^Department of Microbiology, Faculty of Veterinary Medicine, Cairo University, Giza 12211, Egypt; ^2^Department of Zoonoses, Faculty of Veterinary Medicine, Cairo University, Giza 12211, Egypt

## Abstract

In Egypt, the River Red Gum (*Eucalyptus camaldulensis*) is a well-known tree and is highly appreciated by the rural and urban dwellers. The role of *Eucalyptus* trees in the ecology of *Cryptococcus neoformans* is documented worldwide. The aim of this survey was to show the prevalence of *C. neoformans* during the flowering season of *E. camaldulensis* at the Delta region in Egypt. Three hundred and eleven samples out of two hundred *Eucalyptus* trees, including leaves, flowers, and woody trunks, were collected from four governorates in the Delta region. Thirteen isolates of *C. neoformans* were recovered from *Eucalyptus* tree samples (4.2%). Molecular identification of *C. neoformans* was done by capsular gene specific primer *CAP64* and serotype identification was done depending on *LAC1* gene. This study represents an update on the ecology of *C. neoformans* associated with *Eucalyptus* tree in Egyptian environment.

## 1. Introduction

The basidiomycetes yeast of genus * Cryptococcus* includes* C. neoformans*/*C. gattii* species complex, which is composed of two separate species,* C. neoformans* and * C. deneoformans,* and five species within * C. gattii*. The most clinically relevant complex species were recently revised based on phenotypic and genotypic diversity, supported by the presence of distinct and consistent lines, and it proposes to recognize* C. neoformans *var.* grubii* (represented by genotypes VNI and VNII) and* C. neoformans* (VNIII and VNIV) as separate species, as well as five species of* C. gattii* (represented by genotypes VGI, VGII, VGIII, and VGIV) [1, 2].

*C. neoformans* has a worldwide distribution and has been recovered from pigeon droppings (*Columba livia*), urban environments, and soil. Many reports have shown the presence of* C. neoformans *in the hollows of different tree species, proposing that trees play a major role in* Cryptococcus* infection [3, 4]. The most common isolate responsible for this fungal infection is* C. neoformans *var.* grubii* serotype A [1, 5–7].* C. gattii* has been proposed to have a specific ecological association with a number of* Eucalyptus* species [8].

*E. camaldulensis* is a well-known tree in Egypt since it was imported by Mohamed Ali, the Governor of Egypt (1805–1848), for fixing the River Nile banks in the 19th century. It is one of the most widely distributed trees in most of the arid and semiarid areas. This kind of tree exists on almost every roadside in Egypt, but there are no data about its role as potential carrier of fungal elements.

In Egypt, the incidence of* Cryptococcus neoformans* from* Eucalyptus* trees and pigeon droppings has been reported [9]. In this report, the author depended on the conventional differentiation methods to determine* C. neoformans* varieties. There is no recent noticeable information about* the Eucalyptus tree role* in the ecology of* C. neoformans* in Egyptian environment. Therefore, the present study was aimed at determining the possible role of this tree as a potential dispersing source of* C. neoformans *in Delta region's environment.

## 2. Materials and Methods

### 2.1. Study Area and Sampling Collection

The study area in Delta region covered 240 kilometers (150 mi) of Mediterranean coastline of Egypt. A total 311 samples out of 200* Eucalyptus* trees, including leaves, flowers, and woody trunks, were collected from four different governorates in the Delta region (Cairo, Giza, Elmenofia, Al-Sharqia) by the rate of fifty samples for each region (Table 1). The samples were stored on ice in clean, sterile plastic bags and transferred to the Microbiology Department laboratory, Faculty of Veterinary Medicine. The samples were rinsed in sterile distilled water, then immersed in sterile saline solution supplemented with chloramphenicol (10.0 mg/mL), and homogenized with ultrahomogenization for 4 min. The bottle was left for 30 min at room temperature to settle the sediment.

### 2.2. Isolation and Identification

From the supernatant fluid of each homogenized sample, a loopful was streaked onto plates of Sabouraud dextrose agar with chloramphenicol and incubated at 30°C for 48 hours. The colonies suspected to be* C. neoformans* were streaked on* Eucalyptus* leaves agar media [12]. The isolates were identified by classical mycological procedures of* C. neoformans* [13].

### 2.3. Molecular Characterization

#### 2.3.1. DNA Extraction

The yeast cells from SDA slants were collected after 48-hour incubation with sterile PBS. The collected pellets were mixed into a microtube with 500 *μ*L TES (100 mM Tris, pH 8.0, 10 mM EDTA, and 2% SDS); 50–100 *μ*g Proteinase K from an appropriate stock solution was added and then incubated for 30 min (minimum) up to 1 h at 55°–60°C with occasional gentle mixing. The lysate mix salt concentration was adjusted to 1.4 M with 5 M NaCl (=140 *μ*L); 1/10 vol (=65 *μ*L) of 10% CTAB was added and incubated for 30 min at 65°C. The lysate was mixed gently and then incubated for 30 min at 0°C; finally, the mix was centrifuged for 10 min at 4°C, at 15000 rpm. The supernatant was transferred to a 1.5 mL tube followed by the addition of 0.55 vol isopropanol (=510 *μ*L) to precipitate DNA followed by immediate centrifugation for 5 min, at 15000 rpm.

### 2.4. Molecular Identification by Capsular Gene

Detection of* C. neoformans* was done by using specific capsular gene primers* CAP64*. The primers for* CAP64* were designed on the basis of DNA sequences (Table 2) [10].

### 2.5. Molecular Differentiation of Serotypes

This was applied by subjecting genomic DNA of identified strains by* CAP64* gene to multiplex PCR amplification using a set of four primers of the laccase gene (*LAC1*) (Table 2) which were used for differentiating four major serotypes, A, D, B, and C, of* C. neoformans* [11].

## 3. Results

### 3.1. Recovery Rate of* C. neoformans* from* Eucalyptus camaldulensis*

**Figure 1:**
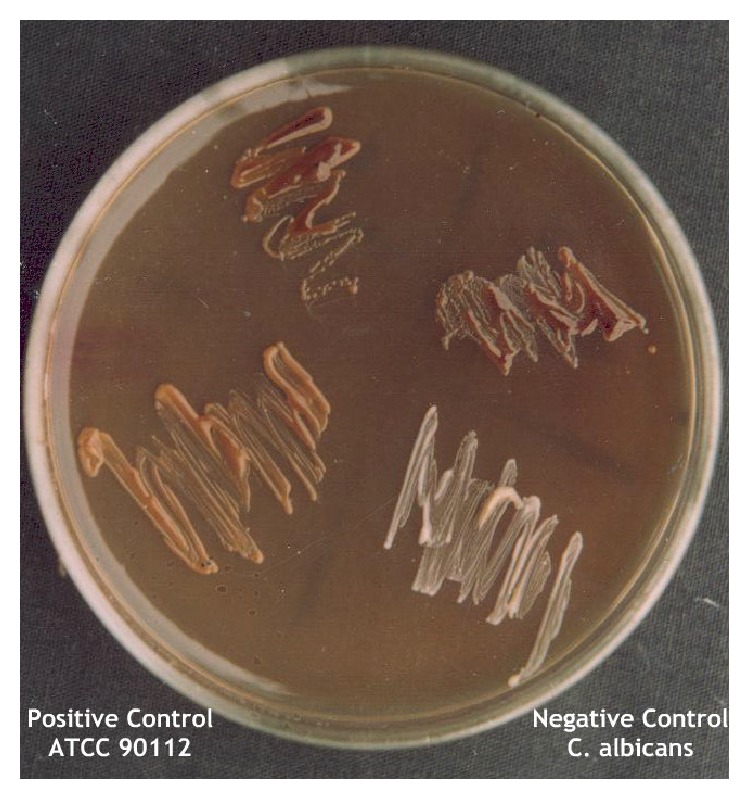
Brown color effect of* C. neoformans *on* Eucalyptus* leaves agar media; white colonies of* C. albicans *negative control and brown pigmented colonies of positive control and environmental isolates.

In this study,* E. camaldulensis* trees acted as a potential refuge for* C. neoformans*. A total of 13 (4.2%)* C. neoformans* isolates out of 311 examined samples in Delta region were recovered during the flowering season of* Eucalyptus* tree. All the recovered* Cryptococcus* isolates were identified as* C. neoformans* strains based on all conventional and physiological characters of* C. neoformans* (Figure 1). Among these, 7 isolates (7.9%) were recovered from 88* Eucalyptus *flowers, 5 isolates (3.6%) were recovered from 138* Eucalyptus* tree leaves, and 1 isolate (1.1) was recovered from 85 woody trunks (Table 3).

### 3.2. Molecular Identification and Differentiation of* C. neoformans*

**Figure 2:**
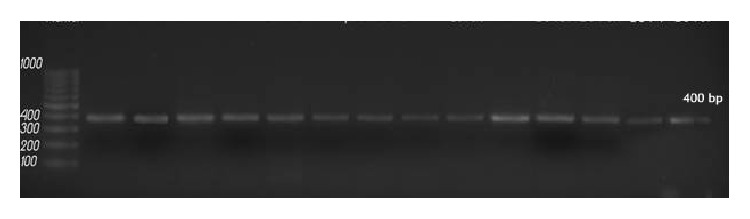
Agarose gel electropheresis of* CAP64* gene specific PCR of all the examined* C. neoformans* isolates with production of amplicons of 400 bp, marker 100 bp DNA ladder (Jena Bioscience).

**Figure 3:**
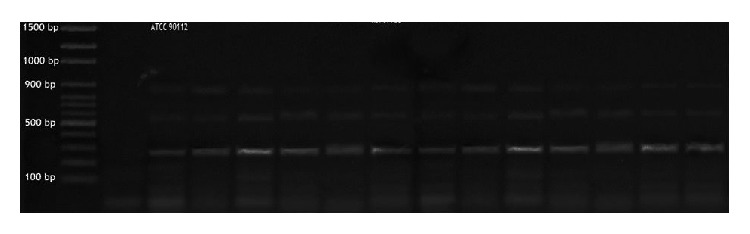
Agarose gel electropheresis of* LAC1* gene specific PCR of all the examined* C. neoformans* isolates with production of amplicons of 250, 760, and 880 bp, marker 100 bp DNA plus ladder (Jena Bioscience).

All tested isolates and reference strain were produced (400 bp) by* CAP64* specific capsular gene primer (Figure 2). Molecular typing of* C. neoformans* isolates was done by four primers for* LAC1* gene (Table 2) which were used for amplification; serotype A strains produced three DNA fragments with sizes of 0.88, 0.76, and 0.25 kb (Figure 3). All tested* C. neoformans* strains were identified as* C. neoformans *var.* grubii *and there are no other serotypes of* C. neoformans *detected.

## 4. Discussion

In Egypt,* Eucalyptus* trees are in abundance mainly as windbreaks and for afforestation of the drains and canals or other watercourses, plus the highways and roads in rural or urban areas.* E. camaldulensis* has a potential role in* C. neoformans *ecology, particularly var.* gattii*. In Australia (home country of* E. camaldulensis*), Ellis and Pfeiffer, 1990 reported the first environmental isolation of* C. gattii* from wood, bark, leaves, and plant debris of* Eucalyptus* trees [14]. Although* Eucalyptus* is present in many of the areas known to have* C. gattii* cryptococcosis, the actual isolation of* C. gattii* from* Eucalyptus* trees is rare outside Australia. Moreover, imported* Eucalyptus* has not been associated with the environmental presence of* C. gattii* in Spain, Central Africa, Canada, Papua New Guinea, Egypt, and Italy [15].

On the African level, the isolation of* C. gattii* from the environment is somewhat limited in comparison to the isolation of* C. neoformans*. Only two cases were recorded in isolation of* C. gattii* from* E. camaldulensis* in African countries, Egypt [9] and Tunisia [16].

In Egypt, earlier report of Mahmoud (1999) [9] depended on canavanine-glycine-bromothymol blue (CGB) agar to determine* C. neoformans* variety, which evoked a high need to investigate the environmental ecology of this fungus, depending on molecular techniques to determine the actual variety of* C. neoformans *in relation to* E. camaldulensis* in order to establish a real surveillance program and applying the preventive measures for this pathogen infection.

This study was applied on* Eucalyptus* trees during the flowering season, as most* C. neoformans* and* C. gattii* reported cases were associated with* Eucalyptus* showing strong seasonality in its occurrence, which coincides with the periods of flowering [17].

The results show that the isolation of* C. neoformans* from* Eucalyptus* flowers is more frequent than from leaves and woody trunk (Table 3). All examined isolates were identified as* C. neoformans *var.* grubii* with a recovery rate of 4.2% of the total examined samples. It is normally the high isolation rate of var.* grubii *as the global distributed isolate responsible for cryptococcal infection [1, 5–7]. Also, it is commonly the recovering of* C. neoformans* from pigeon droppings, soil, and decaying wood in hollow trees [3].

Ambitiously, the present study documents the first record for the isolation of var.* grubii *from* E camaldulensis *leaves, flower, and woody trunks in Egypt. Most of the previous reports stated that* C. grubii* association with* Eucalyptus* trees or other types of trees is interpreted in one sentence: “*C. neoformans* presence might represent fecal contamination by birds inhabiting these trees” [9, 14, 16].

Globally, many reports are recorded for isolation of* C. grubii* from* Eucalyptus* tree parts or other types of trees. In India, more than one report states that* C. grubii *tree association and its distribution differ from each part of tree or season or time of the study. The prevalence of* C. grubii* (5.56%) and* C. gattii* (9.26%) from decayed wood inside trunk hollows of diverse tree species was reported [18].

Recently,* C. grubii* was isolated from the bark of* Eucalyptus* trees followed by flower, bud, fruit, and detritus [19]. The prevalence of* C. grubii* in this study (4.2%) is somewhat near to the rate of Nawange et al.'s (2006) [18] study (5.5%), while the recovery rate of* C. grubii* is the highest from flowers (7.9%), then leaves (3.6%), and finally woody trunks (1.1%) (Table 3).

In sunny countries, C*. neoformans* can escape from lethal effects of sunlight and drying by taking trees as a good natural habitat in the environment because these pathogens can live inside woody debris as well as trunk hollows.The result of the present study highlighted the potential role of tree parts of* E. camaldulensis* in environmental ecology of* C. grubii*.* Eucalyptus* flowers were the best natural habitat and a suitable transporting means for these pathogen infectious propagules in the surrounding environment. Flowering season of* Eucalyptus* tree is mainly in winter and spring from November to February. At this time of year in Egypt, the temperature is slightly low to temperate which gives a potential chance for isolation of this pathogen. The association between* C. grubii* and tree is controlled by many environmental factors including humidity, temperature, and solar radiation [13, 17].

In Egypt, the* Eucalyptus* tree exists almost along every roadside, especially in the Delta region around River Nile and its tributaries. These study results confirm the potential role of* Eucalyptus* trees as a major source for* C. grubii* in Egyptian environment which act as a high risk for immunocompromised patients.

Most of the reported cases of human cryptococcosis were registered as cryptococcal meningitis. Cryptococcal meningitis in Egypt is rarely diagnosed, but this may be due to inadequate investigation rather than absence of definite epidemiological data about the organism in Egypt. More attention should be considered for human cases of unexplained chronic meningitis that is not responding to conventional therapy as* C. neoformans* could be the main cause of such fetal meningitis [20–23].

The only survey for fungal meningitis was done in Egypt at NAMRU-3 during the period of 1998 to 2001 of 1000 CSF samples, where 10* C. neoformans* were recovered at a rate of 0.01%. All isolates belonged to serotype A (*C. neoformans *var.* grubii*) [24]. Recently,* C. neoformans* serotype A is the most common variety in association of pet birds droppings in the Egyptian environment [25].

This study's findings come in the same direction with the previous surveillance of the main causes of cryptococcal infection in Egypt and it confirmed that* C. neoformans *var.* grubii* is the main etiological agent of cryptococcal infection in Egypt.

Conclusively, this is the first record describing isolation of* C. neoformans *var.* grubii *from* E. camaldulensis* in Africa and Egypt. The results highlighted the potential role and risk of* Eucalyptus *tree as a carrier reservoir of one of the high pathogenic fungal elements in Egypt.

## Figures and Tables

**Table 1:** *Eucalyptus* tree collected samples in the Delta region.

Governorate	Tree number	Samples type			Total
		Leaves	Flowers	Wood trunks	
Giza	50	40	25	30	95
Cairo	50	31	13	15	59
Al-Sharqia	50	50	30	30	110
Elmenofia	50	17	20	10	47
Total	200	138	88	85	311

**Table 2:** Primers used in this study.

Primer	Primer sequence 3′-5′	PCR product	Reference
*CAP64*	GCCAAGGGAGTCTTATATGG GCAAAGGGTTCACCAAATCG	400 bp	[10]

*LAC1*	GGAACAGCAACCACACTACTG CATATTGGGTGGCATCTTACTGAGGGA CCAGGGAACATGTTGTTGAC GTTGTGGAAGGCAAAGAAAC	250 bp 760 bp 880 bp	[11]

**Table 3:** Recovery rate and distribution of * C. neoformans * in tested *Eucalyptus* trees sampled in the Delta region.

	Total samples	Giza	Cairo	Al-Sharqia	Elmenofia	Total	Recovery rate
		Number of *C. neoformans *isolates					
Leaves	138	1	1	2	1	5	3.6
Flowers	88	1	1	3	2	7	7.9
Woody trunks	85	1	0	0	0	1	1.1
Total	311	3	2	5	3	13	4.2
